# The Effect of Clinical Decision Prompts in Improving Human Papillomavirus Vaccination Rates in a Multispecialty Practice in a Predominantly Hispanic Population: Quasi-Experimental Study

**DOI:** 10.2196/42890

**Published:** 2023-03-15

**Authors:** Jennifer Molokwu, Melissa Mendez, Christina Bracamontes

**Affiliations:** 1 Department of Family And Community Medicine Texas Tech University Health Sciences Center El Paso El Paso, TX United States; 2 Department of Obstetrics and Gynecology Hospital Corporation of America Las Palmas Del Sol Healthcare El Paso, TX United States; 3 Department of Obstetrics and Gynecology Texas Tech University Health Sciences Center El Paso El Paso, TX United States

**Keywords:** HPV, HPV vaccination, electronic clinical decision support, EMR prompt, clinical, decision, vaccine, pediatrics, age, ethnicity, race, language, immunization

## Abstract

**Background:**

The human papillomavirus (HPV) is implicated in the causal pathway of cancers of the vulva, vagina, penis, cervix, anus, and oropharyngeal region. It is the most common sexually transmitted infection in the United States. Despite the documented safety and effectiveness of the HPV vaccine, rates lag behind those of other vaccines given at the same age.

**Objective:**

Provider recommendation is identified as a robust predictor of HPV vaccine uptake, and physician-prompting is shown to increase the provision of preventive care services in general. Theoretically, providing reminders to providers should increase opportunities for providing HPV vaccine recommendations and therefore affect vaccination rates. The objective of our study was to assess the effectiveness of an electronic medical record (EMR) prompt in improving HPV vaccination rates in an academic clinic setting caring for a predominantly Hispanic border population.

**Methods:**

We used a quasi-experimental design with a retrospective chart audit to evaluate the effect of a clinical decision prompt (CDP) on improving HPV immunization rates in different specialty settings. We introduced an EMR prompt to remind providers to recommend the HPV vaccine when seeing appropriate patients in an obstetrics and gynecology (OBGYN), pediatrics (PD), and family medicine (FM) clinic in a large multispecialty academic group located along the Texas-Mexico border. We assessed HPV vaccination rates in all the departments involved before and after introducing the prompts. Participants included male and female patients between the ages of 9 and 26 years, presenting at the clinics between January 2014 and December 2015.

**Results:**

We reviewed over 2800 charts in all 3 clinics. After adjusting for age, ethnicity, race, type of insurance, preferred language, and clinic, the odds of immunization were 92% (*P*<.001) higher in patients after the prompt implementation of the EMR. In addition, there was an overall statistically significant increase in the overall HPV vaccination completion rates after implementing the CDP (31.96% vs 21.22%; *P*<.001). Again, OBGYN saw the most significant improvement in vaccination completion rates, with rates at follow-up 66.02% higher than baseline rates (*P*=.04). PD and FM had somewhat similar but no less impressive improvements (57.7% and 58.36%; *P*<.001).

**Conclusions:**

Implementing an EMR CDP improved our overall odds of HPV vaccination completion by 92%. We theorize that the decision prompts remind health care providers to discuss or recommend the HPV vaccination during clinical service delivery. CDPs in the EMR help increase HPV vaccination rates in multiple specialties and are a low-cost intervention for improving vaccination rates.

## Introduction

The human papillomavirus (HPV) is implicated in the causal pathway of cancers of the vulva, vagina, penis, cervix, anus, and oropharyngeal region [1,2]. HPV is the most common sexually transmitted infection in the United States [3] and accounts for over 30,000 cancers annually [4]. In addition, persistent infection with oncogenic strains of HPV has been associated with over 90% of cervical cancers [5], with HPV infection also associated with 63% of penile cancers [4,6].

The Federal Drug Administration approved the HPV vaccine in 2006 for use in female individuals aged 9 to 26 years; the indication was expanded 3 years later, in 2009, to include male individuals [7,8]. Despite the proven efficacy of these vaccines in the prevention of persistent HPV infection as well as Cervical Intraepithelial Neoplasia 2+ lesions [9], HPV vaccination uptake has been slow, and rates of initiation and completion still lag behind those of other adolescent vaccines recommended at the same age [10]. Rates of HPV vaccination among adolescents aged 13 to 17 years are approximately 41%, compared with rates for tetanus-diphtheria-acellular pertussis and meningococcal conjugate vaccine at 87.6% and 60%, respectively [11]. Hispanic female participants, especially those living on the US-Mexico border, bear an unequal burden of incident cervical cancer. The cervical cancer mortality rate among female individuals living on the US-Mexico border is the highest in the nation at 5.7/100,000 compared to the national average of 2.4/100,000, age-standardized to the year 2000 population [12]. Most penile cancers (63%) are associated with HPV infection [4], and Hispanic male individuals have the highest incidence in the country at 1.9 per 100,000 compared to 1.1 per 100,000 among non-Hispanic White male participants. [13].

Numerous factors are identified as barriers to the increased uptake of the HPV vaccine, including parental concerns about cost, vaccine safety, potential side effects, and possible promotion or condoning of youth sexual behavior [14-16]. Provider recommendation is identified as a robust predictor of HPV vaccine uptake [17,18]. The acceptability of the HPV vaccine is higher in individuals who received a recommendation from their providers or believed their providers would recommend it [16,19].

Despite the documented efficacy of provider recommendations, reports suggest that providers tend to give weak or inconsistent recommendations for the HPV vaccine compared to other adolescent vaccines [20] and are more likely to portray it as optional rather than routine [21]. Barriers reported by providers include perceived perception of parental hesitancy, poor provider knowledge, concern about the discussion of the sexual mode of transmission, and HPV requiring more time and effort to discuss when compared to other vaccines [22-26].

Dorell et al [27] reported that 66% of parents of unvaccinated adolescents (HPV) said they had not received a recommendation from their providers. Additionally, across the differing specialties, only approximately 50% of providers always recommend the HPV vaccine at visits, pointing to numerous missed opportunities to discuss HPV vaccination [26]. Physician-prompting is shown to increase the provision of preventive care services in general [28]. Theoretically, providing reminders to providers should increase opportunities for providing HPV vaccine recommendations and therefore affect vaccination rates. However, the evidence of the effect of prompts on improving adolescent vaccine rates has not been consistent, with some studies showing no difference [29] and others showing a significant improvement in adolescent vaccine rates with electronic prompting [30]. The objective of this study was to assess the effectiveness of an electronic medical record (EMR) prompt in improving HPV vaccination rates in an academic clinic setting caring for a predominantly Hispanic border population. Evaluating the significance of this low-cost intervention in a high-risk population can help inform structural changes to improve HPV vaccination rates in clinical settings with limited resources.

## Methods

### Settings

We carried out our study at an academic medical center near the US-Mexico border. The Medical Center comprises 13 clinical departments with over 200,000 patient visits a year. The center is also home to training for medical and nursing students, residents, and fellows. The City of El Paso has a population of over 700,000, with approximately 80% of Hispanic origin, and a median household income of US $32,000 [31].

### Population

We selected the 3 departments that were most involved in the care of individuals in the HPV vaccination age range. These were the family medicine (FM), pediatrics (PD), and obstetrics and gynecology (OBGYN) departments. These were also the only departments that stocked the HPV vaccine in their clinics.

All patients aged 9 to 26 years who received care at these 3 clinics during the period of interest were eligible. For the department of PD, we excluded their specialty clinics (oncology, cardiology, endocrinology, gastroenterology, and nephrology).

### Study Design

We conducted a quasi-experimental design with a retrospective chart audit to evaluate the effectiveness of a clinical decision prompt (CDP) in improving HPV immunization rates. In addition, we provided 1 live educational lecture for each department separately to increase our knowledge of the HPV disease process and the HPV vaccine product for our physicians. The same attending OBGYN physician gave the lecture to each department and included residents, attending physicians, and any midlevel providers. Table 1 contains the characteristics of clinical providers for descriptive purposes. These lectures were held during 3 different periods in the final quarter of 2014. We introduced EMR prompts in January 2015.

**Table 1:** Characteristics of clinical providers participating in the educational session.

Characteristics				Clinical specialty						Overall (N=84)			*P* value	
				PD^a^ (n=32)		OBGYN^b^ (n=22)		FM^c^ (n=21)						
Age (years), mean (SD)				35.28 (11.7)		37.89 (10.14)		38.2 (10.96)	37.06 (10.8)			.62		
Years of practice, mean (SD)				18.33 (14.61)		10.55 (10.99)		11.7 (14.74)	12.07 (12.9)			.38		
**Gender, n (%)**														.19
	Male		17 (48.6)		7 (20)		11 (31)		35 (47)					
	Female		13 (34)		15 (39)		10 (26)		40 (53)					
**Race, n (%)**														.02
	White		12 (30)		17 (43)		11 (28)		41 (49)					
	Other races or unknown		20 (57)		5 (14)		10 (29)		43 (51)					
	**Hispanic, n (%)**													.001
		Yes	6 (20)		9 (30)		15 (50)		30 (40)					
		No	24 (56)		13 (30)		6 (14)		45 (60)					
**Years of practice, n (%)**														.15
	≤10 years		3 (19)		7 (44)		6 (38)		18 (21)					
	11-20 years		0 (0)		2 (50)		2 (50)		4 (5)					
	>20 years		3 (43)		2 (29)		2 (29)		7 (8)					
	No experience or in training or residency		26 (54)		11 (23)		11 (23)		55 (65)					

^a^PD: pediatrics.

^b^OBGYN: obstetrics and gynecology.

^c^FM: family medicine.

### Data Abstraction

All departments use the same EMR. We received a list of all individual visits per department for the year in question. We conducted a random audit of 10% (3120/31,200) of the charts of patients within the age range of 9 to 26 years who visited these clinics in the calendar year January to December 2014 to assess our baseline HPV vaccination in 3 departments: OBGYN, PD, and FM. We used a random number generator to obtain a random sample of the patients based on our sample size calculator. We assessed that obtaining 10% of the clinic visits for the year would get us to our appropriate number per sample size calculation. Individually selected charts were abstracted by volunteer students using our chart abstraction tool. Volunteers were instructed in all charts to check the vaccine flow sheet, orders tab, and nurse and clinician office visits. Patients had completed the series if all 3 doses were documented in their chart or if providers noted historical completion during the clinic visit. Historical vaccination status was documented in a chart for patients with shot records or immunization records indicating they received the vaccines elsewhere. We repeated this process for the data audit in the post intervention data for the calendar year 2015.

We calculated our sample size based on a national estimate of the prevalence of HPV vaccination [11]. We powered our study to detect at least a 10% change in our HPV vaccination rates after implementing our CDP. Based on these estimates, 2460 participants (1500 female and 960 male participants) would be required to achieve greater than 90% power to detect a difference between group proportions using a 2-sided Fisher exact test at a 1% significance level. We estimated the sample size using PASS 12 (NCSS LLC) [32].

We instituted a CDP in our EMR to flag patients aged 9 to 26 years whenever they came in for office visits to encourage providers to discuss HPV vaccination and vaccinate as appropriate. The prompt appeared once after the provider accessed the patient's chart. Providers could ignore this prompt and continue their clinic visit if they so decided. The prompt was set to lapse once the clinic staff documented the HPV vaccination in the patient's chart. Following the initiation of the electronic prompt, we carried out a second chart audit on another 1230 randomly selected charts for the 12 months starting in January 2015.

### Ethical Considerations

Before beginning the study, we obtained approval from the Texas Tech University Health Science Center El Paso Institutional Review Board (reference number 059324), and the study was determined to be exempt. Participant information was obtained via abstraction from patient records conducted as a chart audit. No individual patient identifier was stored in the data set used for analysis. Since this was done as part of the evaluation of a clinical process, separate patient consent was not required.

### Analysis

Age was collected as a continuous variable in years from the participant chart. Race in medical records is categorical: Black, White, Asian, American Indian or Alaska Native, and Hawaiian or Pacific Islander. Due to small numbers and unstable estimates in racial categories, race was dichotomized as White participants and non-White participants. Ethnicity is documented in the chart as a categorical variable (Hispanic participants vs non-Hispanic participants).

We described continuous variables using the mean and SD, while categorical variables were described using frequencies and proportions. We used chi-square statistics to assess the differences in study arms for categorical variables. In contrast, for continuous variables, we used the *t* test and the Wilcoxon rank-sum test (for skewed variables). Using a logistic regression model, we assessed the adjusted and unadjusted association between baseline factors and HPV immunization in the pre- and postintervention arms. The variables adjusted for were age, ethnicity, race, type of insurance, preferred language, and clinic. For patients in the OBGYN clinic, we also adjusted for sexual activity since this information was only collected in the OBGYN clinic and may affect the acceptability of HPV vaccines [33]. We excluded the age of first intercourse and the age of HPV vaccination since these variables were not consistently documented and there was not enough data to assess. Therefore, we considered it statistically significant, with *P* values less than 5%, and performed all analyses using SAS V. 9.4 (SAS Institute).

## Results

We reviewed 2,851 charts (we oversampled male participants in the other clinics to ensure we represented males well, especially given that the OBGYN department was bound to have only female patients). Patients in the postintervention cohort were older (age in years 17.6 vs 16.5, *P*<.001), more likely to be female (784/1290, 60.8% vs 745/1561, 47.8%, *P*<.001), and more likely to be Hispanic in origin (1045/1290, 81% vs 1208/1561, 77.4%, *P*=.02), and for the OBGYN department alone, 9% (27/272) and 2.9% (7/232) reported being sexually active (*P*=.004; see Table 2).

There was an overall statistically significant increase in the overall HPV vaccination completion rates after implementing the CDP (412/1289, 31.96% vs 331/1560, 21.22%, *P*<.001). OBGYN saw the greatest improvement in vaccination completion rates, with rates at follow-up 66.02% higher than baseline rates (*P*=.04). PD and FM had somewhat similar but no less impressive improvements, 57.7% and 58.36% (*P*<.001). Rates at baseline were higher in the PD department when compared to FM and obstetrics (221/659, 33.5% vs 88/651, 13.5% vs 22/250, 8.8%), and this difference was maintained even after the intervention (see Table 3).

After adjusting for age, ethnicity, race, type of insurance, preferred language, and clinic, the odds of immunization completion were 92% higher in all patients after the CDP implementation (odds ratio [OR] 1.92, 95% CI 1.59-2.32). Factors significantly associated with receipt of vaccination include having private insurance (OR 3.16, 95% CI 1.76-5.65), attending PD and FM clinics (OR 4.01, 95% CI 2.8-5.76 and OR 1.7, 95% CI 1.18-2.45, respectively), and being of Hispanic origin (OR 1.43, 95% CI 1.07-1.89; see Table 4).

**Table 2:** Patient baseline characteristics comparing pre- and postintervention cohorts.

Variables		Preintervention (n=1561)	Postintervention (n=1290)	*P* value	
Age (years), mean (SD)		16.5 (5.75)^a^	17.6 (5.46)^a^	<.001	
Age (years) at first sexual intercourse, mean (SD)		17.4 (2.16)^a^	16.68 (1.86)^a^	.17	
Number of sexual partners, median (IQR)		2 (1-4)^a^	2 (1-4)^a^	.60^a^	
Age (years) vaccine was received, mean (SD)		11.69 (3.42)^a^	12.19 (3.17)^a^	.05	
**Insurance, n (%)**					.09
	Private insurance	365 (25.8)	282 (23.7)		
	Medicaid or CHIP^b^	919 (65.0)	818 (68.7)		
	Hospital discount program, clinic discount program, breast and cervical cancer screening program, or other	131 (9.3)	90 (7.6)		
**Ethnicity, n (%)**					.02
	Hispanics	1208 (77.4)	1045 (81.0)		
	Non-Hispanics	352 (22.6)	245 (19.0)		
**Race, n (%)**					.41
	White	1187 (76.0)	963 (74.7)		
	Non-White	374 (23.96)	327 (25.35)		
**Language preferred**					.72
	English	1024 (65.9)	855 (66.5)		
	Spanish or other	531 (34.2)	430 (33.5)		
**Gender**					<.001
	Female	745 (47.8)	784 (60.8)		
	Male	815 (52.2)	505 (39.2)		
**Is the patient sexually active?^c^, n (%)**					.004
	No	7 (2.9)	27 (9.0)		
	Yes	232 (97.1)	272 (91.0)		
**Which valent vaccine was given?, n (%)**					.46
	Bivalent (ie, Cervarix)	149 (45.4)	137 (34.2)		
	Quadrivalent (ie, Gardasil)	96 (29.3)	105 (26.2)		
	9-valent	83 (25.3)	159 (39.7)		

^a^Wilcoxon sum rank test.

^b^CHIP: Children’s Health Insurance Program.

^c^Data collected only in obstetrics clinic.

**Table 3:** HPV vaccination completion rates by clinics^a^.

Has the patient had immunizations for HPV^b^ (HPV vaccination rates): for all FM^c^, PD^d^, and OBGYN^e^ clinics		Preintervention (n=1560), n (%)	Postintervention (n=1289), n (%)	*P* value	
**Response for all FM, PD, and OBGYN clinics**					<.001
	No	1229 (78.78)	877 (68.04)		
	Yes	331 (21.22)	412 (31.96)		
**Response for on PD clinic only**					<.001
	No	438 (66.46)	235 (47.09)		
	Yes	221 (33.54)	264 (52.91)		
**Response for FM clinic only**					<.001
	No	563 (86.48)	378 (78.59)		
	Yes	88 (13.52)	103 (21.41)		
**Response for OBGYN clinic only**					.04
	No	228 (91.2)	263 (85.39)		
	Yes	22 (8.8)	45 (14.61)		

^a^Completion is defined as receiving 3 doses of the HPV vaccine.

^b^HPV: human papillomavirus.

^c^FM: family medicine.

^d^PD: pediatrics.

^e^OBGYN: obstetrics and gynecology.

**Table 4:** Adjusted and unadjusted association between HPV vaccination completion and study arm for all clinics^a,b^.

Variables (dependent variable: HPV^c^ immunizationyes)		Unadjusted association			Adjusted association	
		OR^d^ (95% CI)	*P* value	OR (95% CI)		*P* value
**Study arm**						
	Before implementation	1	N/A^e^	1		N/A
	After implementation	1.74 (1.47-2.07)	<.001	1.92 (1.59-2.32)		<.001
Age (in years)		0.96 (0.95-0.98)	<.001	1.01 (0.99-1.03)		.33
**Race**						
	White	1	N/A	1		N/A
	Non-White	0.76 (0.62-0.93)	.007	1.04 (0.82-1.32)		.85
**Insurance**						
	Medicaid or CHIP^f^	2.23 (1.26-3.93)	.006	1.69 (0.92-3.13)		.17
	Private insurance	7.23 (4.24-12.33)	<.001	3.16 (1.76-5.65)		<.001
	UMC, Texas Tech Discount, breast and cervical cancer screening program, or other	1	N/A	1		N/A
**Clinic**						
	PD^g^	5.28 (3.99-7)	<.001	4.01 (2.8-5.76)		<.001
	FM^h^	1.49 (1.11-2.01)	.009	1.7 (1.18-2.45)		<.001
	OBGYN^i^	1	N/A	1		N/A
**Ethnicity**						
	Hispanics	2.16 (1.7-2.74)	<.001	1.43 (1.07-1.89)		.006
	Non-Hispanics	1	N/A	1		N/A
**Language preferred**						
	English	1	N/A	1		N/A
	Spanish or other	2.34 (1.97-2.78)	<.001	1.38 (1.12-1.70)		.003
**Gender**						
	Female	1	N/A	N/A		N/A
	Male	1.06 (0.90-1.26)	.47	N/A		N/A

^a^Completion is defined as receiving 3 doses of the HPV vaccine.

^b^Adjusted for age, ethnicity, race, type of insurance, preferred language, and clinic.

^c^HPV: human papillomavirus.

^d^OR: odds ratio.

^e^N/A: not applicable.

^f^CHIP: Children’s Health Insurance Program.

^g^PD: pediatrics.

^h^FM: family medicine.

^i^OBGYN: obstetrics and gynecology.

## Discussion

### Principal Findings

Implementing an EMR CDP improved our overall odds of completing HPV vaccination by 92%. This result differed from a previous randomized controlled trial that did not find increased vaccine uptake in adolescent vaccines using EMR prompts [29]. This previous study was a large multiclinic study using primarily pediatric and FM clinics and evaluating all adolescent vaccines. There was no difference in vaccination status for all vaccines and HPV between those clinics that initiated a prompt and those centers that did not. We theorize that the difference in population demographics may have played a role (only 11% to 19% of participants were Hispanic). However, we found studies that agreed with our findings and showed an increase in vaccination following the introduction of CDPs [34]. Ruffin et al [30] reported increased HPV vaccination rates using comparative community clinics. This study population was also not similar to ours and consisted mostly of White and African participants.

We theorize that the decision prompts remind health care providers to discuss or recommend the HPV vaccination during clinical service delivery. Studies show that while a strong recommendation is more effective, discussing the HPV vaccine also increases HPV vaccine rates [35]. Our study adds to this body of knowledge, confirming that low-cost interventions such as CDPs significantly improve HPV vaccination rates (at least in the short term) in a primarily Hispanic cohort.

After adjustment, the odds of HPV vaccination remained significantly higher for pediatric and FM clinics and were highest for PD at baseline. This higher rate for HPV vaccination in PD is consistent with reports showing higher initiation and completion rates in pediatric clinics compared to FM and other specialties [36]. We theorize that this may be due to systems set in place (vaccines are routinely given in pediatric clinics) and the possibility that pediatricians are more invested in vaccinations in general and may provide more robust recommendations. In addition, studies have shown that the consistency and strength of recommendation are higher among pediatric practitioners than FM practitioners. This finding may partially account for the higher vaccination initiation and completion rates in these clinics [37]. This difference in the strength of recommendation opens up a target area of focus for intervention with FM and OBGYN providers who are likely to see older adolescents and young adults who may have missed the HPV series when they were younger.

The strengths of our study include the large number of patient charts that were audited across 3 different clinics. As a result, our pre- and postintervention groups were not identical, eliminating any duplication of charts. In addition, we have a large, predominately Mexican-American population, which is underrepresented in the literature. Other studies have found that physicians can ignore prompts or skip over them due to “prompt fatigue” [38]. To limit “prompt fatigue,” we restricted this study to 1 year. However, we think it is important to look forward to the future to see if the gains made will persist.

### Limitations

There are limitations to this study. Our study was not a randomized controlled trial of HPV prompts versus no prompts. Therefore, we were limited by using the same EMR in all clinics we evaluated, and it would have been technically challenging to randomize by clinic. The differences in the baseline rates by clinic also made randomization by clinic not feasible. Other possible confounding factors include changes in awareness about the vaccine over time and variations in rates over the year. We accounted for these potential differences by reviewing the same periods (January to December) in both years. In addition, we provided lectures for each department separately to increase the knowledge of the HPV disease process and the HPV vaccine product for our physicians. Our patient population is 85% Mexican-American and has been shown in previous studies to be open to HPV vaccination, with reports as high as 66% vaccination rates in El Paso County [30,37]. In the FM clinic and PD clinics, an electronic vaccination record within the EMR documents historical vaccine administration. All vaccines given in the 3 clinics are recorded electronically in the vaccine administration record. However, the OBGYN clinic does not consistently record historical vaccines administered in an electronic vaccination flowsheet and instead may record vaccine history within the medical note, usually within the History of Present Illness, creating a poor tracking record of the vaccines that may have skewed actual vaccine rates in this clinic. To correct this, the guideline for chart audits included reviewing all clinic notes in all the clinics for documentation of HPV vaccination during the year in question.

We carried out our study at an academic institution along the Texas-Mexico border. We did not include community-based clinics and private physician offices. Thus, our findings may not apply to all populations across the United States. Our patient population also has a high level of uninsured or underinsured patients, which may have affected our before and after HPV vaccination rates. We also did not include data on the timeliness of vaccination for all 3 doses of the HPV vaccine for each patient.

### Conclusions

Our study shows that a simple, inexpensive EMR prompt for vaccination and provider education on HPV disease and the HPV vaccine increased our vaccination rates in all 3 clinical settings. Prompts in the EMR are a low-cost intervention for improving vaccination rates and may have an unmeasurable impact on our patients and their risk of cervical, anal, vaginal, and oropharyngeal cancers.

Future directions for improving HPV vaccination rates may include better tracking of vaccine status among patients in the EMR for an accurate rate. Medical staff may require further education, including standardizing provider counseling points, to promote vaccination to all eligible patients. Clinic staff may need training on the importance of screening for unvaccinated patients to alert the physicians to offer the vaccine. Explicitly targeting certain patients, such as those coming in for late vaccination past the 9- to 11-year-old start time, male patients, and perhaps postpartum patients may also increase the HPV vaccine uptake rates. Providing free vaccines and patient visits through grants in the patient's neighborhood or school may increase the HPV vaccine rate.

## Abbreviations

CDP: clinical decision prompt
EMR: electronic medical record
FM: family medicine
HPV: human papillomavirus
OBGYN: obstetrics and gynecology
PD: pediatrics

## Data Availability

The data sets generated during and analyzed during the current study are not publicly available due to the source being chart audits from patient data. However, they are available from the corresponding author upon reasonable request.
